# Epigallocatechin-3-gallate conjugated with selenium nanoparticles prevents neurological complications in rats exhibiting schizophrenia-like behaviors

**DOI:** 10.3389/fphar.2025.1680380

**Published:** 2025-10-15

**Authors:** Hadeer M. Gamal El-Deen, Amina E. Essawy, Nema A. Mohammed, Mohamed S. Abdelfattah, Ayah S. Fathalla, Manal F. El-khadragy, Ahmed E. Abdel Moniem

**Affiliations:** ^1^ Euro-Mediterranean Program in Neuroscience and Biotechnology, Faculty of Science, Alexandria University, Alexandria, Egypt; ^2^ Zoology Department, Faculty of Science, Alexandria University, Alexandria, Egypt; ^3^ Chemistry Department, Faculty of Science, Helwan University, Cairo, Egypt; ^4^ Zoology and Entomology Department, Faculty of Science, Helwan University, Cairo, Egypt; ^5^ Department of Biology, College of Science, Princess Nourah bint Abdulrahman University, Riyadh, Saudi Arabia; ^6^ Al-Ayen Scientific Research Center, Al-Ayen Iraqi University, Nasiriyah, Iraq

**Keywords:** epigallocatechin gallate and selenium nanoparticles, schizophrenia, social isolation, oxidative stress, neuroinflammation, neuromodulation, apoptosis

## Abstract

**Introduction:**

The main catechin in green tea is a flavonoid called (-)-epigallocatechin 3-gallate (EGCG) that possesses significant biological and pharmacological properties. In a rat model of schizophrenia, we examined the neuroprotective and antipsychotic properties of EGCG conjugated with selenium nanoparticles (EGCG-SeNPs) against neurological complications induced by social isolation, including significant behavioral and neurochemical dysfunctions that mimic the symptoms of schizophrenia in it.

**Methods:**

Male rats (21–23 days old) were divided into two groups: the social rearing (SR) group and the social isolation-reared (SIR) group (one rat per cage). The experiment lasted for eight weeks. For the last 2 weeks, rats in both SR and SIR were assigned to saline, EGCG (50 mg/kg), sodium selenite (0.5 mg/kg), EGCG-SeNPs (0.5 mg/kg), and risperidone (2.5 mg/kg) treated groups. At the end of the experiment, all rats were subjected to behavioral tests, and the prefrontal cortex tissues from each group were analyzed for oxidative stress parameters, proinflammatory cytokines, neurochemicals, apoptotic markers, and histopa‐thological changes.

**Results:**

EGCG‐SeNPs treatment improved the behavior of rats, significantly decreased the levels of malondialdehyde, nitric oxide, and the pro-inflammatory mediators TNF-alpha, IL-1beta, and NF-κB, raised the expression of antioxidant glutathione, superoxide dismutase, glutathione reductase, and catalase, enhanced monoaminergic and cholinergic transmission, and restored the excitatory-inhibitory amino acid imbalance. Additionally, EGCG-SeNPs improved the histopathological changes in the prefrontal cortex, upregulated the expression oe Bcl-2, and downregulated the expression of the anti-apoptotic Bax and caspase-3.

**Discussion:**

These encouraging anti-inflammatory, anti-oxidative, anti-apoptotic, and neuromodulatory activities suggest that EGCG-SeNPs might serve as a naturally derived antipsychotic agent.

## 1 Introduction

Schizophrenia is a chronic and persistent illness that affects approximately 1% of the population during lifetime, and its pathophysiology and etiology remain unknown (Marder and Cannon, 2019). Several hypotheses have been proposed to explain the physiopathology of this disease, including the dopamine hypothesis and glutamate hypothesis (McCutcheon et al., 2020). The symptoms of schizophrenia are divided into three categories: negative symptoms, which indicate functioning deficits and include social disassociation and a decline in motivation; positive symptoms, which represent errors of normal functioning and include delusion and hallucinations, paranoia, and agitation; and cognitive symptoms, which include deficiencies in memory, focus, learning, and executive skills (El-Sisi et al., 2016). One of the main causes of schizophrenia’s symptoms is the malfunctioning of the prefrontal cortex (PFC), which affects the ability to change the target of perception (Szlachta et al., 2017).

Antipsychotic drugs used to treat schizophrenia are divided into two types, known as typical (first-generation drugs) and atypical (second-generation drugs). Typical antipsychotic medications cause extrapyramidal adverse effects, and although atypical antipsychotic drugs, such as risperidone, are associated with fewer extrapyramidal side effects, they frequently induce weight gain (Chokhawala and Stevens, 2023), which contributes to insulin resistance, hyperlipidemia, and increased risk of prediabetes and cardiovascular diseases (Boyda et al., 2013). Additionally, antipsychotic medicines are ineffective in treating negative symptoms and cognitive impairment (Ahmed et al., 2018; Jamilian and Ghaderi, 2021).

Because of the brain’s low amounts of antioxidant enzymes and its high oxidative metabolic activity, the brain is especially susceptible to the harmful effects of reactive oxygen species (Da Silveira Paulsen et al., 2012). Multiple researchers have documented higher concentrations of nitric oxide (NO) and malondialdehyde (MDA) in the peripheral tissues, cerebrospinal fluid, and plasma of individuals with schizophrenia, accompanied by decreased levels of antioxidants (Coughlin et al., 2012).

Green tea’s active polyphenol, epigallocatechin-3-gallate (EGCG), has attracted considerable interest because of its potential health benefits, including anti-inflammatory, anti-oxidative, anticancer, radical-scavenging, metal-chelating, and anti-apoptotic properties (Singh et al., 2010). EGCG enhances memory and the learning process by reducing the levels of oxidative indicators such as NO and MDA (Baluchnejadmojarad and &Roghani, 2011).

The human body contains selenium (Se), which is widely distributed throughout its tissues and organs and is a necessary trace element for sustaining the body’s regular physiological processes. Se can engage in free radical reactions through its antioxidant effect, thereby reducing the damage caused by oxidative stress (Xie et al., 2018; Habbab et al., 2019). Selenium is essential to many different brain activities. It is involved in memory, cognition, motor function, coordination, and the pathophysiology of brain disorders such as Parkinson’s disease, epilepsy, and Alzheimer’s disease (Solovyev, 2015; Yuan et al., 2020). In several neurological diseases, selenium treatment modifies oxidative stress, inflammatory parameters, and apoptotic indicators (Demirci et al., 2016; Zheng et al., 2019).

Recently, the biomedical field has gradually expanded its application studies of selenium nanoparticles (SeNPs). SeNPs are a selenium product that has minimal toxicity and great biological activity, making it suitable for use as a medicinal agent or drug delivery system. The potential harm to organisms can be decreased by using SeNPs. Compared to elemental selenium, SeNPs have superior biological activity, a higher absorption rate, and a greater absorption capacity. They have demonstrated antioxidant, antibacterial, antiviral, and anticancer properties in biological applications (Xiao et al., 2023).

In the present study, we aimed to investigate the possible antipsychotic effects of EGCG conjugated with SeNPs (EGCG–SeNPs) against social isolation-induced schizophrenia by assessing oxidative, inflammatory, and apoptotic profiles, along with neurochemical and histological alterations in the prefrontal cortex of rats.

## 2 Materials and methods

### 2.1 Drugs and chemicals

EGCG was purchased from Assaha Wa Aljamal Co. (United States). The synthesis of EGCG–SeNPs was performed as follows: 17.6 mg of sodium selenite (Na_2_SeO_3_) was dissolved in 10 mL of distilled water and mixed with 458.37 mg of EGCG (corresponding to 0.5 mg of sodium selenite loaded onto 13 mg of EGCG) under magnetic stirring for 24 h.

### 2.2 Characterization of EGCG–SeNPs

To evaluate the particle size distribution and zeta potential of SeNPs, dynamic light scattering (DLS) measurements were performed using a Zetasizer Nano ZN (Malvern Panalytical Ltd., United Kingdom) at 25 °C and a scattering angle of 173°.

### 2.3 Experimental animals

Male Wistar albino rats (50–60 g weight and 21–23 days old) were obtained from the Nile Valley Center for Agricultural and Animal Production, Cairo, Egypt. The animals were housed under standard laboratory conditions in wire polypropylene cages (12-h light/dark cycle; 25 °C ± 2 °C). Standard diet and water were given *ad libitum*. All experimental procedures and animal handling were confirmed to the guidelines approved by the Alexandria University Institutional Animal Care and Use Committee (ALEXU-IACUC), a member of the International Council for Laboratory Animal Science (ICLAS) (approval no. AU04-206-25-06-2024).

### 2.4 Induction of schizophrenia and study design

The schizophrenia model was induced by social isolation rearing. Rats were assigned to either the social isolation-reared rats (SIR, one rat per cage) or the social rearing group (SR, 3–4 rats for each cage) after weaning, and the experiment was conducted for 8 weeks (Möller et al., 2013).

For exploration of the anti-schizophrenic activity of SeNPs and EGCG, 60 male Wistar rats were used and divided into 10 experimental groups, with 6 rats in each group (n = 6), and treated as follows during the last 2 weeks (7^th^ and 8^th^ weeks):

Groups I and II (SR and SIR control groups): rats received daily oral and intraperitoneal (i.p.) doses of 0.5 mL of normal saline (0.9% NaCl).

Groups III and IV (SR and SIR EGCG-treated groups): rats received a daily oral dose of EGCG (50 mg/kg) (Li et al., 2020) and a daily (i.p.) normal saline dose.

Groups V and VI (SR and SIR sodium selenite-treated groups): rats received a daily oral dose of sodium selenite (0.5 mg/kg) (Omairi et al., 2022) and a daily (i.p.) normal saline dose.

Groups VII and VIII (SR and SIR EGCG–SeNPs-treated groups): rats received a daily oral dose of EGCG–SeNPs (0.5 mg/kg) and a daily (i.p.) normal saline dose.

Groups IX and X (SR and SIR risperidone-treated groups): rats were injected (i.p.) with risperidone (2.5 mg/kg) (Sedky et al., 2022) and received a daily (i.p.) normal saline dose.

**FIGURE 1:**
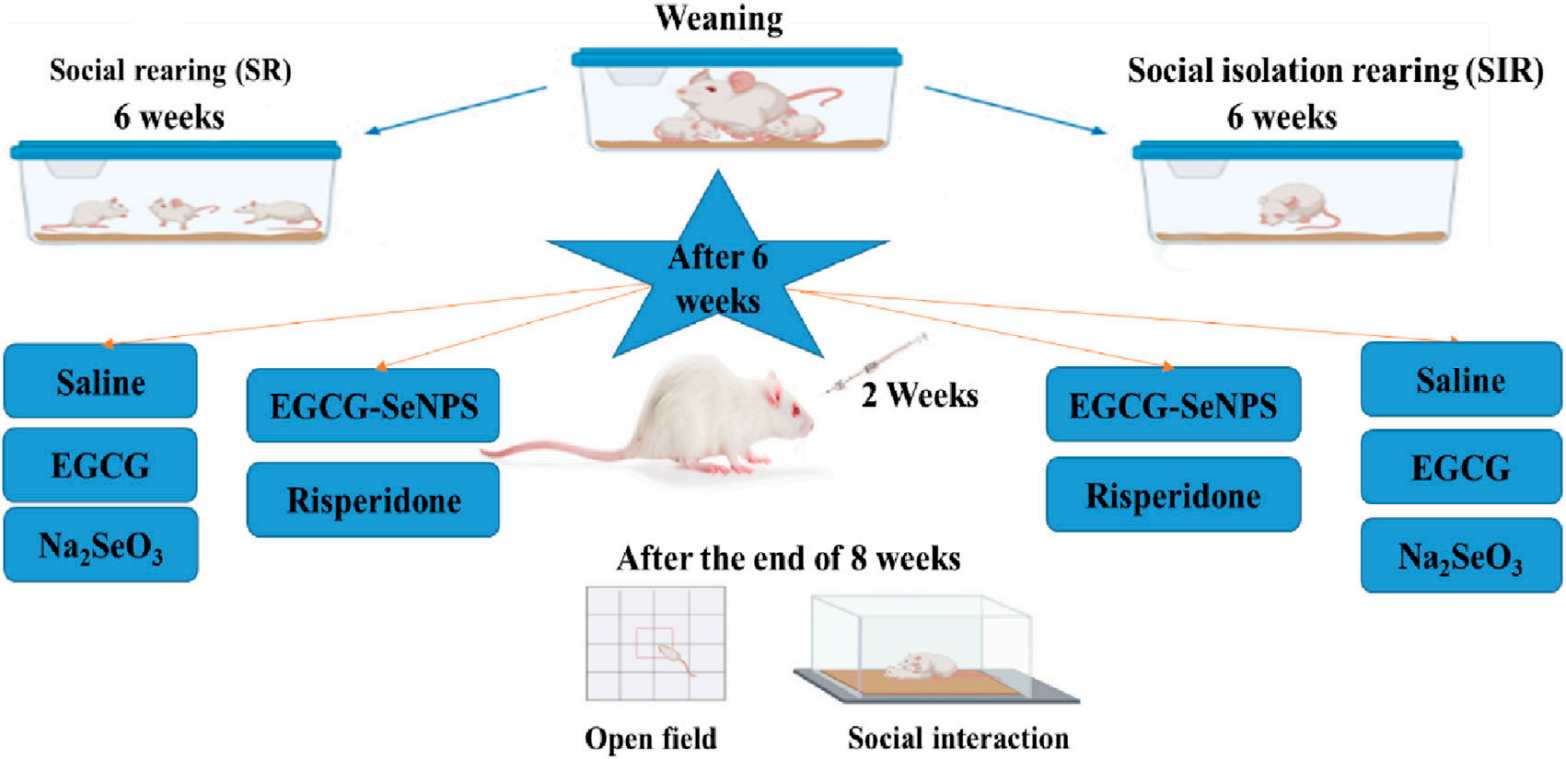
Schematic illustration representing the experimental design applied in this study.

A schematic representation of the experimental design and experimental groups is illustrated in Figure 1.

### 2.5 Behavioral evaluation

The open-field test and the social interaction test were conducted at the end of the experimental period (8 weeks).

#### 2.5.1 Open-field test

The animals’ curiosity was assessed using the open-field test. There were 16 equal-sized squares (60 × 60 × 60 cm) in the open-field area. The behavior of the rats was recorded for 3 min after a 1-min acclimatization period. The number of squares crossed for velocity estimation and the number of grooming and rearing were observed manually (Mahmoud et al., 2025).

#### 2.5.2 Social interaction test

A social interaction test was used to evaluate negative symptoms. Two rats from each group were kept in a cage for 10 min. The number of social contacts, the duration of each contact, and the latency to the first interaction were recorded manually to assess the social behavior of each animal pair (Nawwar et al., 2022).

### 2.6 Sampling and preparation of prefrontal cortex homogenate

Twenty-four hours after behavioral testing, all rats were anesthetized via intraperitoneal administration of ketamine (100 mg/kg) and xylazine (10 mg/kg) and then euthanized by cervical dislocation. The brain tissues were scraped off, and the prefrontal cortex was rapidly removed, washed with isotonic saline, and prepared for biochemical, molecular, and histopathological studies. For biochemical and molecular analysis, prefrontal cortex tissues were homogenized in ice-cold phosphate-buffered saline (10 mM; pH 7.4) and centrifuged at 3,000 *g* for 10 min, and the supernatants were stored at −80 °C.

### 2.7 Determination of oxidant/antioxidant markers

Ellman’s reagent was used to determine the GSH levels in the prefrontal cortex. At 412 nm, the yellow chromogen was determined (Ellman, 1959). NO levels in the prefrontal cortex region were evaluated by measuring dye production at 540 nm after the addition of the Griess reagent (Green et al., 1982). MDA was measured using the thiobarbituric acid method to indicate the level of lipid peroxidation (Ohkawa et al., 1979).

The activity of the antioxidant enzymes, including catalase (CAT), superoxide dismutase (SOD), glutathione peroxidase (GPx), and glutathione reductase (GR), was evaluated in the prefrontal cortex according to the methods described by Aebi, (1984), Nishikimi et al. (1972), Paglia and Valentine (1967), and Goldberg et al. (1983), respectively.

### 2.8 Determination of neurochemical parameters

To evaluate the prefrontal cortex’s neurochemical parameters, commercial enzyme-linked immunosorbent assay (ELISA) kits were used to measure the level of corticosterone (Cat. No. MBS761865), brain-derived neurotrophic factor (BDNF) (Cat. No. E-EL-H0010), glutamate (Cat. No. KA 1909), GABA (Cat. No. MBS7612758), dopamine (Cat. No. ENZ-KIT188-001), and serotonin (Cat. No. ADI-900–175). Using the method outlined by Ellman et al. (1961), the prefrontal cortex activity of acetylcholinesterase (AChE) was measured.

### 2.9 Determination of pro-inflammatory cytokines

For estimating pro-inflammatory cytokines TNF-α (MyBioSource, Inc., United States; catalog number: MBS2507393) and IL-1β (RayBiotech Life, Inc., United States; catalog number: ELR-IL-1B), ELISA kits were used, following the guidelines provided by the manufacturer.

### 2.10 Determination of apoptotic proteins (Bax, Bcl-2, and caspase 3)

For measuring prefrontal cortex apoptotic proteins Bax (CUSABIO, United States; catalog number: CSB-EL002573RA), Bcl-2 (Vector Laboratories, Inc., United States; catalog number: LS-F4135) and caspase 3 (Elabscience, United States; catalog number: E-EL-R0160), ELISA kits were also used. All tests were carried out in compliance with the guidelines provided by the manufacturer.

### 2.11 Determination of NF-κB and NMDAR mRNA expression using real-time PCR

RNA was extracted from the prefrontal cortex using the TRIzol method. Using a Cellixi-cDNA synthesis kit, the first-stranded cDNA was formed. The three technical replicates were subsequently amplified with Power SYBR Green and assessed using the Applied Biosystems (7500) device. PCR was performed under the following conditions: one cycle at 95 °C for 10 min, followed by 40 cycles at 95 °C for 15 s, annealing at 57 °C for NF-κB and 60 °C for NMDAR for approximately 30 s, and a final extension at 72 °C for 30 s. Following amplification, each sample’s cycle counts at the linear amplification threshold (Ct) for the reference gene GAPDH were calculated. The comparative Ct method was then used to calculate relative gene expression (Pfaffl, 2001). NF-κB and NMDAR mRNA expressions were estimated. The primer sequences used for RT-PCR are presented in Table1.

**TABLE 1:** Primer sequences used for RT-PCR.

Gene	Forward primer	Reverse primer
NF-κB	TCC​TTT​CGG​AAC​ACT​GGG​CAA​A	AGG​TAT​GGG​CCA​TCT​GTT​GAC
NMDAR	ATGCCGACCTGACCTGAG	TCA​CTG​GTC​TTG​CTG​GTC​C

### 2.12 Histopathological estimation

After fixation of prefrontal cortex tissue samples in 10% neutral buffered formalin, they were dehydrated, embedded in paraffin, and sectioned (4–5 μm). Tissue sections were deparaffinized in xylene, rehydrated through an ethanol series, stained with hematoxylin and eosin, and examined using light microscopy.

### 2.13 Docking interaction study

The main target of both typical and atypical antipsychotic medications is the D2 dopamine receptor (DRD2) (Wang et al., 2018). The effect of EGCG on D2 dopamine receptors was studied. The maestro of Schrödinger’s Glide’s Standard Precision software was used to conduct the docking test. The DRD2–risperidone and DRD2–EGCG structures indicate the structural basis for the effects of risperidone and EGCG at DRD2. The protein 3D structure was obtained from the Protein Data Bank (6cm4) Structure of the D2 dopamine receptor bound to the atypical antipsychotic drug risperidone.

After downloading the ligands EGCG and risperidone from PubChem, they were prepared using the LigPrep tool. Then, the determination of the active side of the protein was performed using the Glide tool for receptor grid generation. The binding energy of the ligand was estimated. After selecting the appropriate DRD2 sequence, protonation was performed, and the partial charges were then computed.

### 2.14 Statistical analysis

Data were analyzed using the statistical program GraphPad Prism version 8.4.3. The data were provided as the mean ± standard deviation (SD). The data were analyzed using one-way ANOVA, and the significance level was set at *p*-values <0.05.

## 3 Results

### 3.1 Characterization of EGCG–SeNPs

**FIGURE 2:**
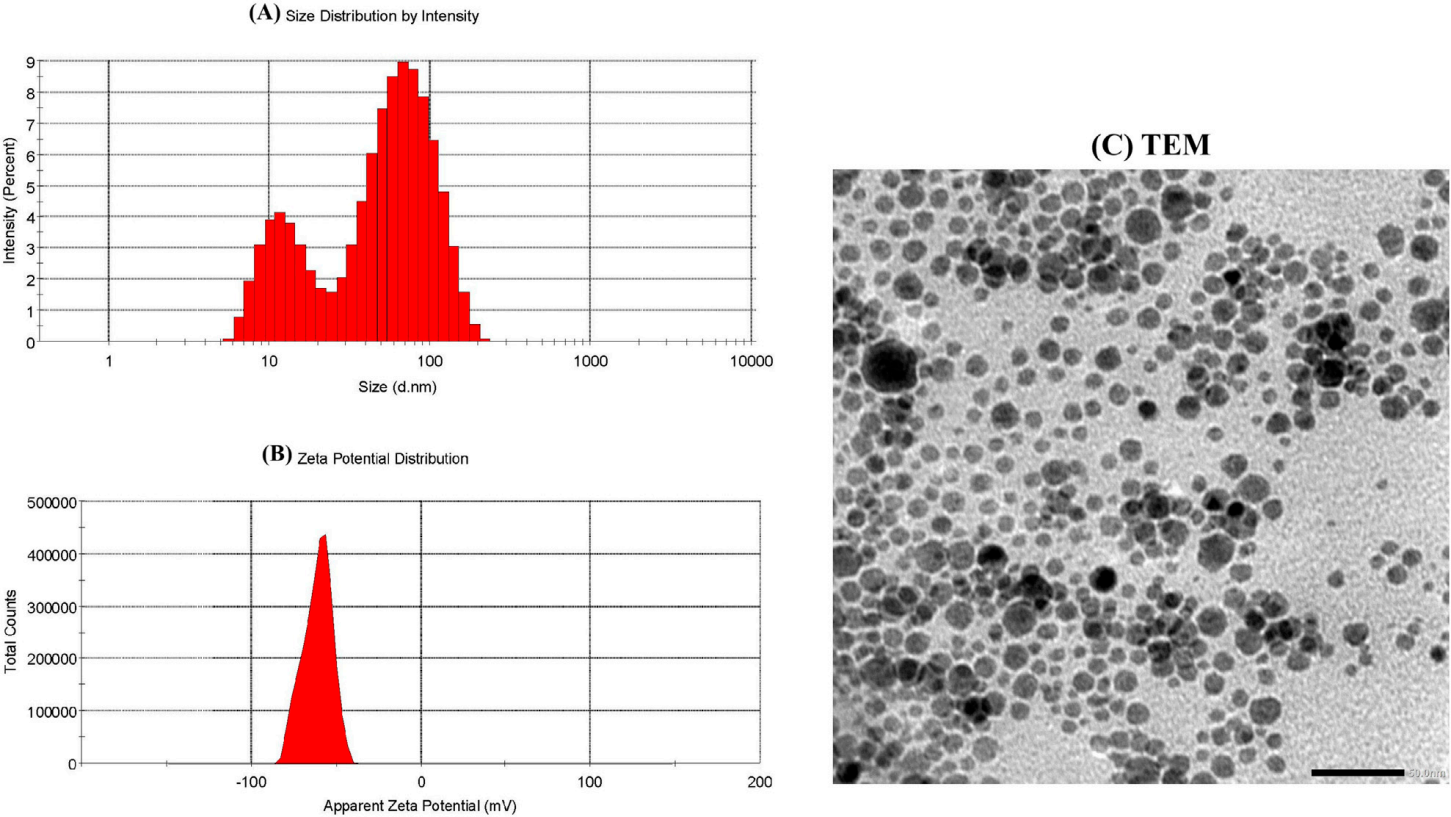
Characterization of EGCG–SeNPs. **(A)** Size distribution of EGCG–SeNPs by intensity. **(B)** Zeta potential distribution of EGCG–SeNPs. **(C)** TEM images of SeNPs recorded at 50–100 nm.

DLS was used to measure the SeNPs’ zeta potential, along with their particle size and size distribution. The results demonstrated that EGCG–SeNPs were characterized with an average diameter of 32.19 nm (Figure 2A) and a mean zeta potential of −61 mV (Figure 2B). When the size, shape, morphology, and aggregation of SeNPs were examined using transmission electron microscopy (TEM), the results showed that the particles were evenly distributed and exhibited spherical to nearly quasi-spherical geometries, with edge lengths ranging from 50 to 100 nm (Figure 2C).

### 3.2 Effect of EGCG–SeNP treatment on the performance of male rats in the open-field test

**FIGURE 3:**
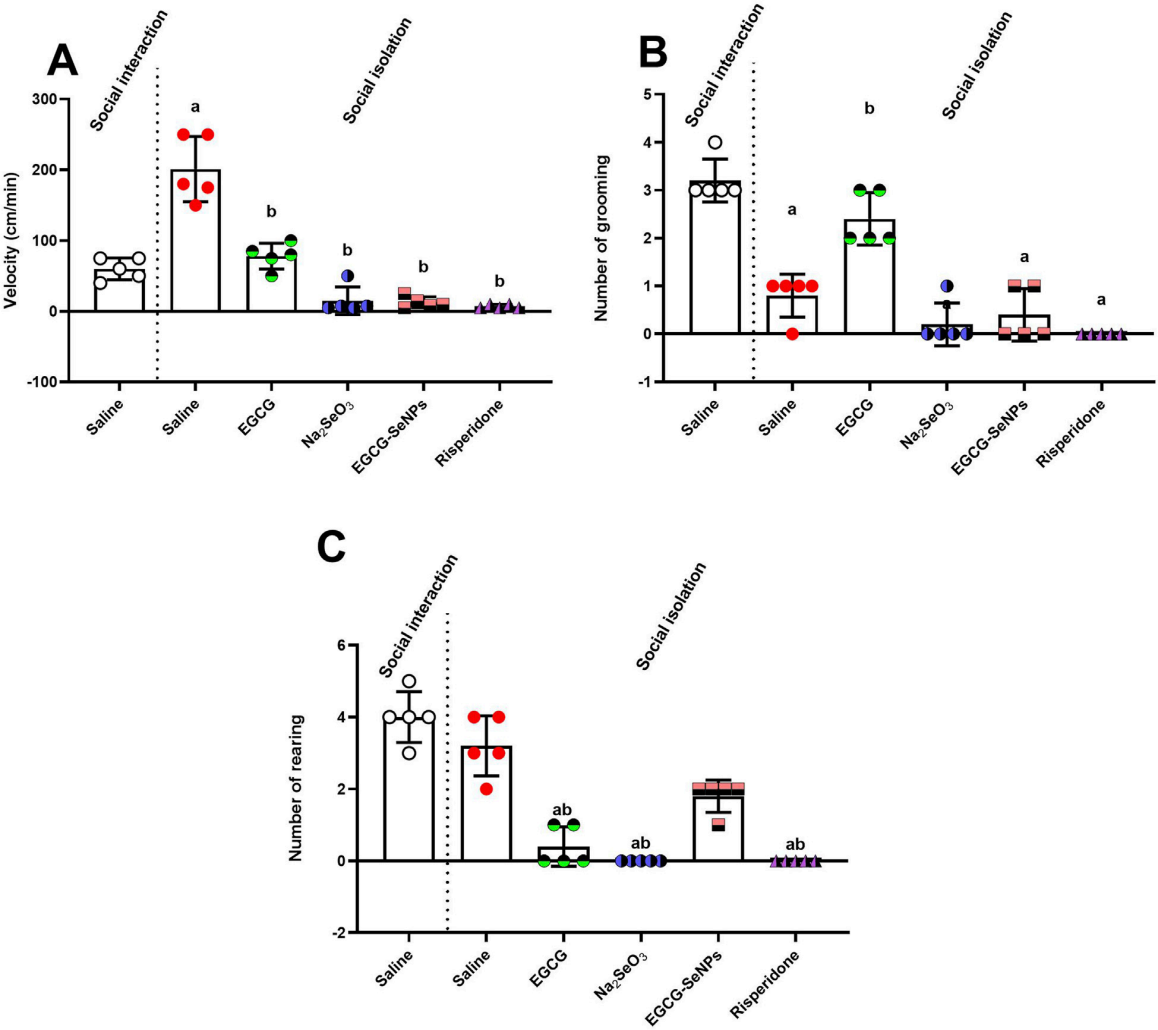
Effects of epigallocatechin-3-gallate (EGCG), sodium selenite (Na_2_SeO_3_), EGCG–SeNPs, and risperidone on locomotor activity in socially isolated rats: **(A)** velocity, **(B)** number of grooming episodes, and **(C)** number of rearing episodes. ^a,b^ denote significant differences (*p* < 0.05) compared to the untreated control and socially isolated groups, respectively. All data are presented as the mean ± SD.

Figure 3A shows that social isolation significantly [*p* < 0.0001; F (5, 24) = 51.86] increased the velocity in male rats compared to the socially interacted control group. However, socially isolated rats treated with EGCG, Na_2_SeO_3_, EGCG–SeNPs, or risperidone exhibited a significant reduction in hyperactivity compared to untreated socially isolated rats.

Furthermore, a significant [*p* < 0.0001; F (5, 24) = 43.37] decrease in the amount of grooming was observed in untreated socially isolated rats compared to their controls (Figure 3B). However, animals treated with Na_2_SeO_3_, EGCG–SeNPs, or risperidone showed a significant decrease in the number of grooming episodes compared to the socially isolated group, whereas treatment with EGCG significantly increased the number of grooming episodes. The number of rearings was slightly [*p* < 0.0001; F (5, 24) = 52.64] reduced in the case of social isolation compared to the control (Figure 3C) and significantly decreased with the other treatments compared to the schizophrenic group.

### 3.3 EGCG–SeNP treatment enhances social deficiencies in social interaction tests

**FIGURE 4:**
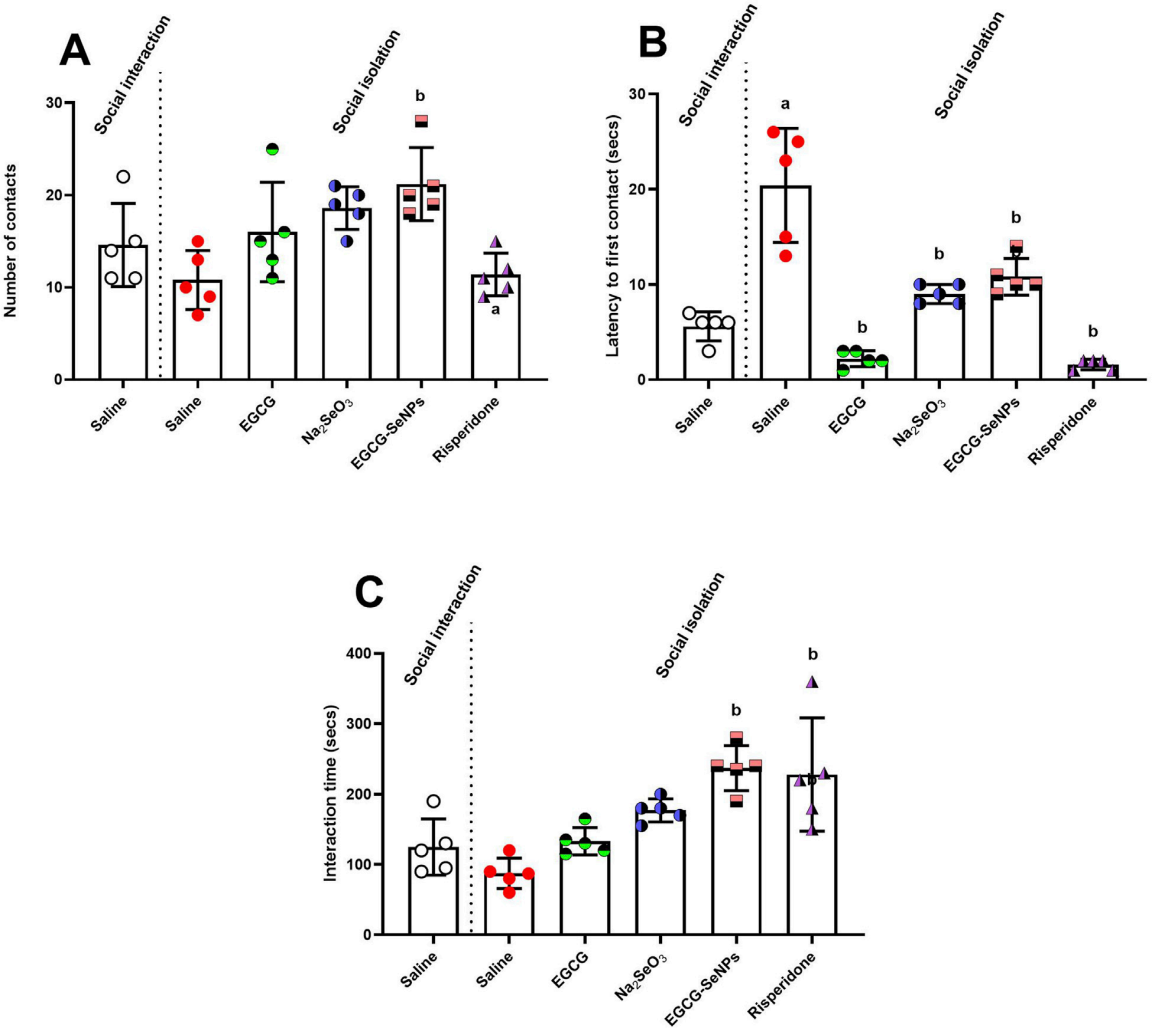
Effects of epigallocatechin-3-gallate (EGCG), sodium selenite (Na_2_SeO_3_), EGCG–SeNPs, and risperidone on social interaction in socially isolated rats: **(A)** Number of contacts, **(B)** latency to first contact, and **(C)** the interaction time of socially isolated rats. ^a,b^ denote significant differences (*p* < 0.05) compared to the untreated control and socially isolated groups, respectively. All data are presented as the mean ± SD.

Figure 4 shows that social isolation induces social deficits, as observed by significantly increased latency to first contact between the animals and insignificantly decreased number of contacts and interaction time compared to socially interacted rats. The number of contacts [*p* = 0.0013, F (5, 24) = 5.737] significantly increased with EGCG–SeNP treatment compared to that in the schizophrenic group. Moreover, the latency to first contact [*p* < 0.0001; F (5, 24) = 33.22] was significantly reduced by all treatments. The interaction time increased [*p* < 0.0001; F (5, 24) = 10.54] with Na_2_SeO_3_, risperidone, and EGCG–SeNPs, while EGCG–SeNP was the best treatment for increasing the duration of communication.

### 3.4 Effect of EGCG–SeNP treatment on the oxidative damage in the prefrontal cortex of socially isolated rats

**FIGURE 5:**
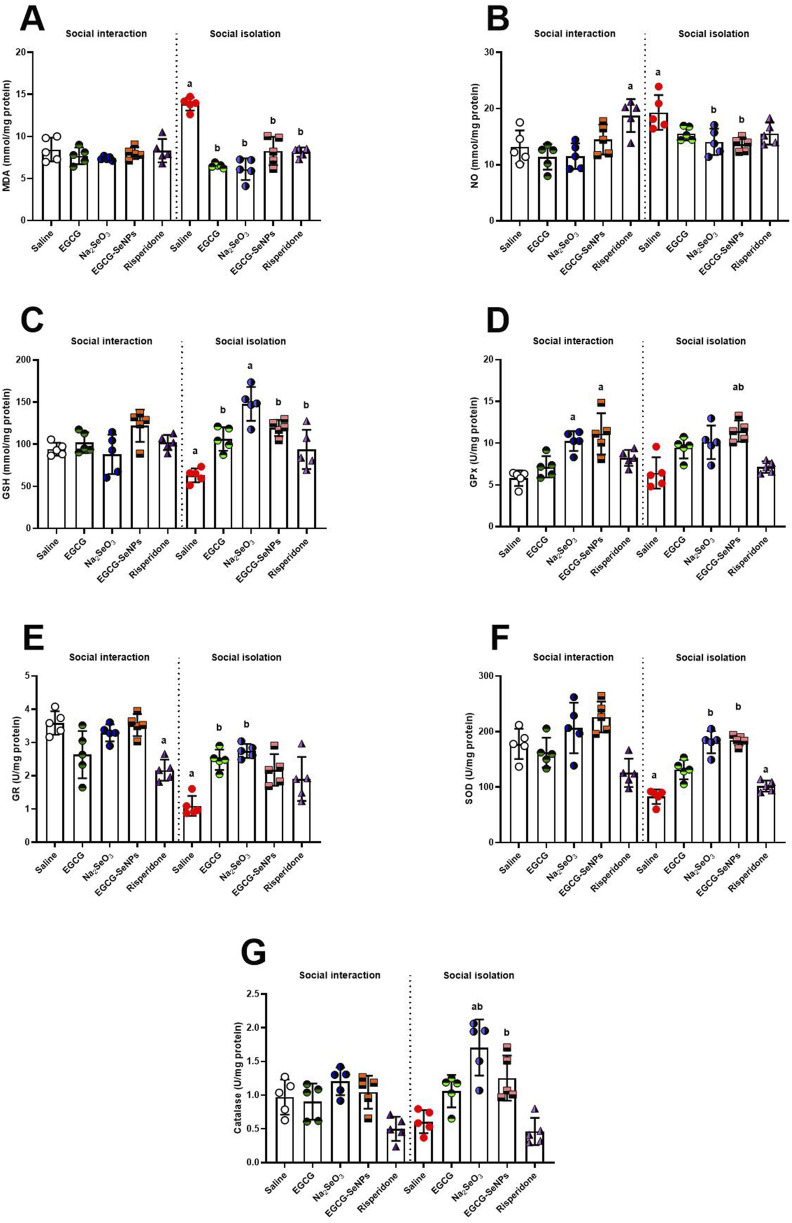
Effects of epigallocatechin-3-gallate (EGCG), sodium selenite (Na_2_SeO_3_), EGCG–SeNPs, and risperidone on the levels of **(A)** malondialdehyde (MDA), **(B)** nitric oxide (NO), **(C)** glutathione (GSH), **(D)** glutathione peroxidase (GPx), **(E)** glutathione reductase (GR), **(F)** superoxide dismutase (SOD), and **(G)** catalase (CAT) in the prefrontal cortex tissue of socially isolated rats. ^a,b^ denote significant differences (*p* < 0.05) compared with the untreated control and isolated groups, respectively. All data are presented as the mean ± SD.

The prefrontal cortex redox status was assessed by estimating the level of oxidative stress and antioxidant markers (Figures 5A–G). Social isolation induces an oxidative stress state that is typified by significantly elevated MDA levels [*p* < 0.0001; F (9, 40) = 20.44] and a significant increase in NO production [*p* < 0.0001; F (9, 40) = 6.303]. Moreover, social isolation resulted in a significant decrease in the levels of GSH [*p* < 0.0001; F (9, 40) = 10.28], GR [*p* < 0.0001; F (9, 40) = 16.84], and SOD activity [*p* < 0.0001; F (9, 40) = 17.71], an insignificant increase in the activities of GPx [*p* < 0.0001; F (9, 40) = 9.231], and an insignificant decrease in the activities of CAT [*p* < 0.0001; F (9, 40) = 10.59] compared to the socially interacted control group. In line with expectations, all administered treatments, particularly EGCG–SeNPs and Na_2_SeO_3_, were able to return the balance between pro-oxidants and cellular antioxidants compared to the schizophrenic (untreated) group.

### 3.5 Effect of EGCG–SeNP treatment on neuroinflammation in the prefrontal cortex of socially isolated rats

**FIGURE 6:**
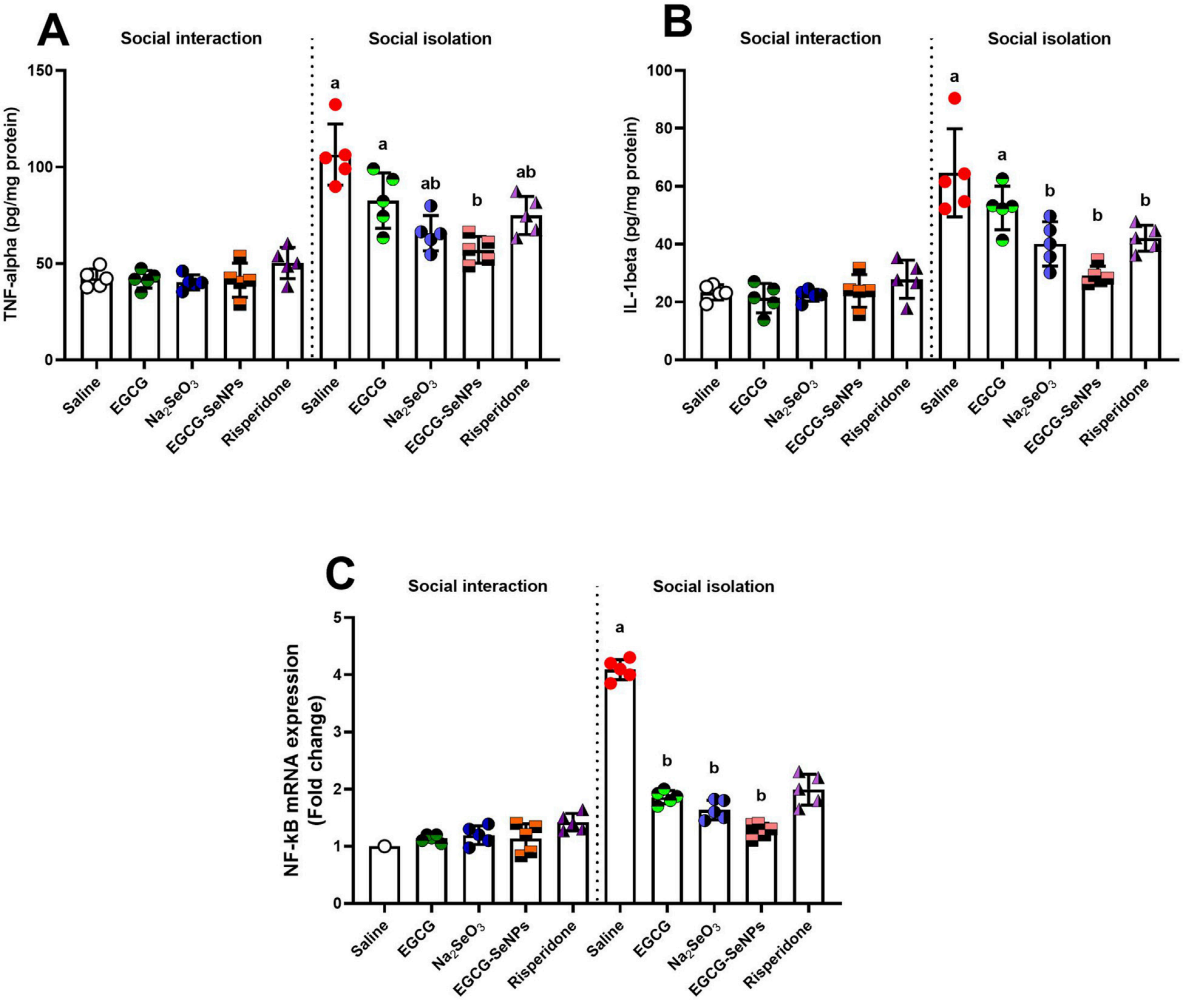
Effects of epigallocatechin-3-gallate (EGCG), sodium selenite (Na_2_SeO_3_), EGCG–SeNPs, and risperidone on the levels of **(A)** TNF-α, **(B)** IL-1β, and **(C)** NF-κB mRNA expression in the prefrontal cortex of socially isolated rats**.** ^a,b^ denote significant differences (*p* < 0.05) compared with the untreated control and isolated groups, respectively. All data are presented as the mean ± SD.

To explore the possible anti-inflammatory effects of EGCG–SeNPs in response to social isolation-induced neuroinflammation, the TNF-α and IL-1β levels and the NF-κB mRNA expression were measured. All pro-inflammatory biomarkers tested in the prefrontal cortex showed a significant increase in the social isolation group compared to the control group [TNF-α (*p* < 0.0001, F (9, 40) = 27.56), IL-1β (*p* < 0.0001, F (9, 40) = 22.20), and NF-κB mRNA expression (*p* < 0.0001, F (9, 36) = 125.3)] (Figure 6). The level of IL-1β significantly decreased after treatment with Na_2_SeO_3_, risperidone, and EGCG–SeNPs and insignificantly decreased with EGCG, compared to the schizophrenic group. The levels of TNF-α and the mRNA expression of NF-κB significantly decreased with all treatments, compared to the schizophrenic group.

### 3.6 Effect of EGCG–SeNP treatment on neurochemical levels in the prefrontal cortex of socially isolated rats

**FIGURE 7:**
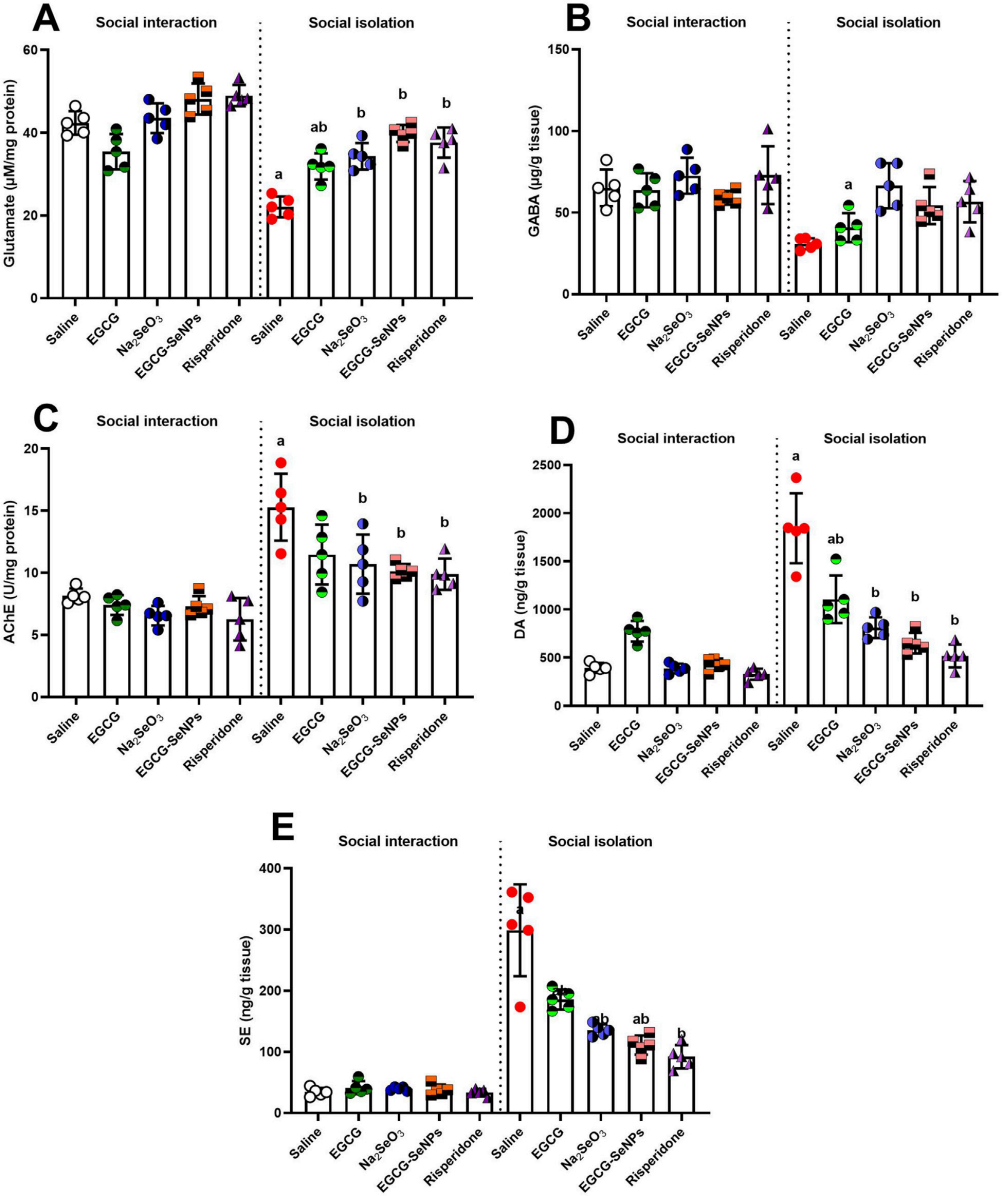
Effects of epigallocatechin-3-gallate (EGCG), sodium selenite (Na_2_SeO_3_), EGCG–SeNPs, and risperidone on the levels of neurochemicals, including **(A)** glutamate, **(B)** GABA, **(C)** AChE, **(D)** dopamine (DA), and **(E)** serotonin (SE), in the prefrontal cortex of socially isolated rats**.** ^a,b^ denote significant differences (*p* < 0.05) compared with the control and isolated groups, respectively. All data are presented as the mean ± SD.

To assess the impact of EGCG–SeNP treatment on the level of neurotransmitters in socially isolated rats, we analyzed prefrontal cortex levels of glutamate, GABA, AChE, dopamine, and serotonin (Figures 7A–E). Socially isolated rats exhibited a significant decrease in the levels of glutamate [*p* < 0.0001, F (9, 40) = 31.33] and GABA [*p* = 0.0017, F (2.541, 10.16) = 11.54] and a significant elevated level of AChE [*p* < 0.0001, F (9, 40) = 14.84], dopamine [*p* < 0.0001, F (9, 40) = 42.02], and serotonin [*p* < 0.0001, F (9, 40) = 54.71] compared to the socially interacted group. Conversely, the levels of AChE, dopamine, and serotonin were significantly decreased with all treatments compared with the schizophrenic group. On the other hand, a significant increase in glutamate levels was observed in all treatments, while a significant increase in GABA levels was observed after treatment with sodium selenite (Na_2_SeO_3_) and risperidone, and an insignificant increase in EGCG–SeNPs and EGCG.

### 3.7 Effect of EGCG–SeNPs on BDNF and corticosterone levels in socially isolated rats

**FIGURE 8:**
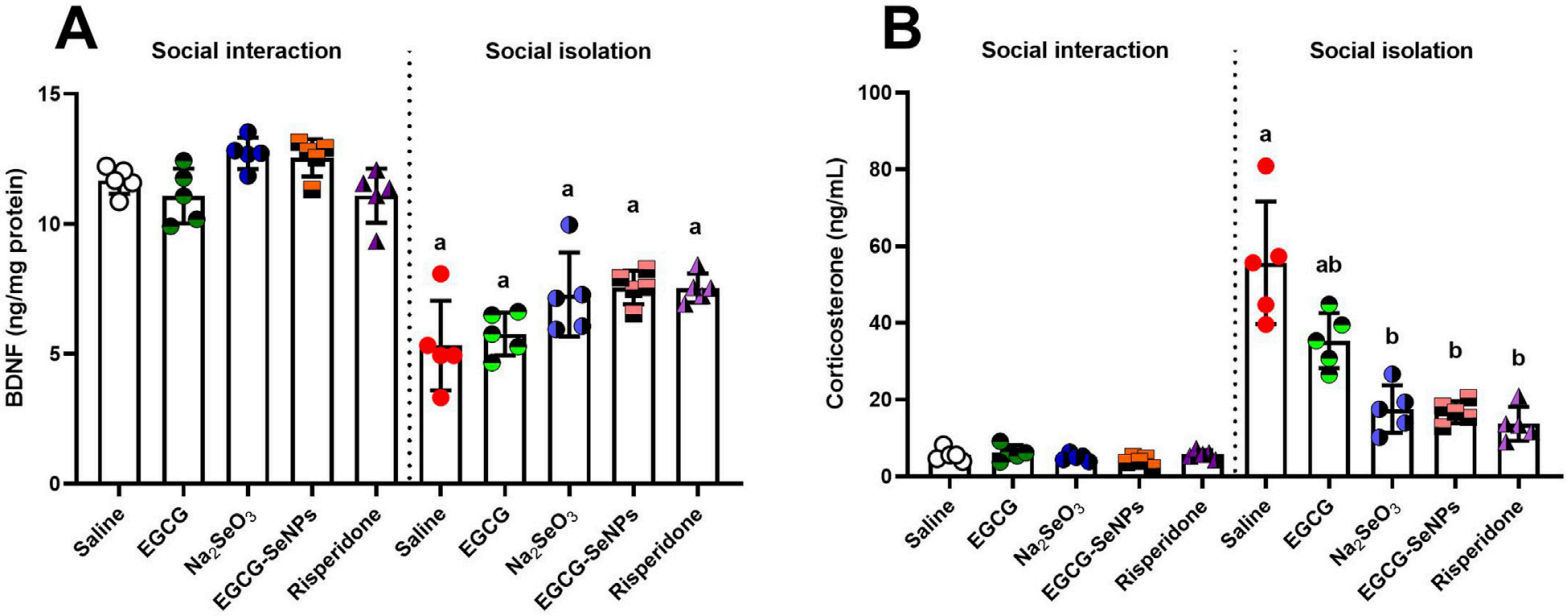
Effects of epigallocatechin-3-gallate (EGCG), sodium selenite (Na_2_SeO_3_), EGCG–SeNPs, and risperidone on **(A)** BDNF and **(B)** corticosterone levels in the prefrontal cortex of socially isolated rats**.** ^a^^,^^b^ denote significant differences (*p* < 0.05) compared with the control and isolated groups, respectively. All data are presented as the mean ± SD.

A significant decrease [*p* < 0.0001; F (9, 40) = 39.03] in the level of BDNF was observed in the socially isolated rats compared to that in the control group. However, an insignificant increase in the level of BDNF was noticed in socially isolated treated groups (EGCG and Na_2_SeO_3_) and significantly elevated levels in EGCG–SeNPs and risperidone, compared to those in the schizophrenic group (Figure 8A). In socially isolated rats, there was a significant increase in the level of corticosterone [*p* < 0.0001; F (9, 40) = 36.78] compared to the control group. Following the 2-week treatments, the level of corticosterone significantly decreased compared to that in the schizophrenic group (Figure 8B).

### 3.8 EGCG–SeNP treatment modulates neuronal loss and regulates neuronal maintenance in prefrontal cortical tissue of socially isolated rats

**FIGURE 9:**
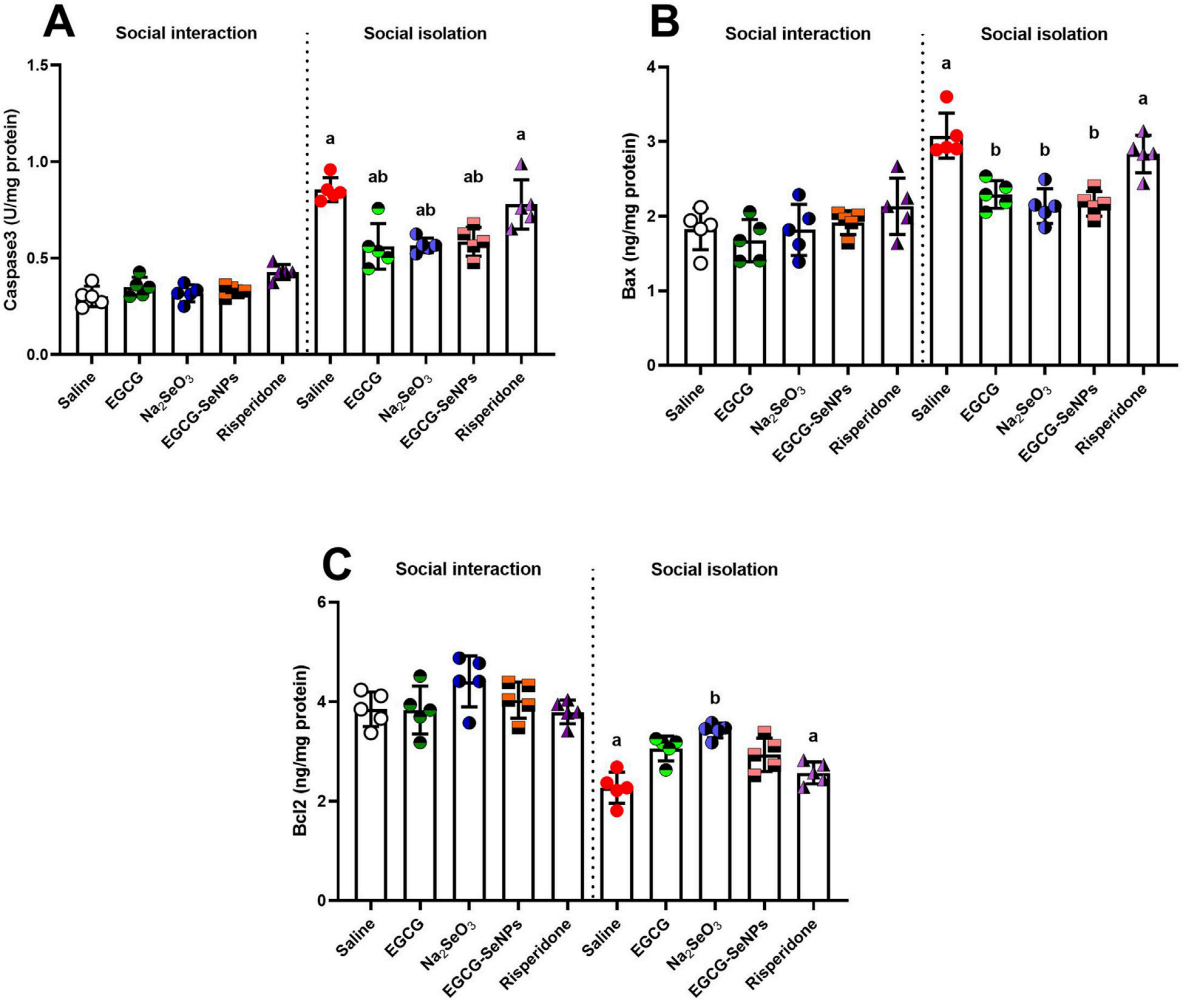
Effects of epigallocatechin-3-gallate (EGCG), sodium selenite (Na_2_SeO_3_), EGCG–SeNPs, and risperidone on the levels of apoptosis markers, including **(A)** caspase-3, **(B)** Bax, and **(C)** Bcl-2, in the prefrontal cortex of socially isolated rats. ^a,b^ denote significant differences (*p* < 0.05) compared with the control and isolated groups, respectively. All data are presented as the mean ± SD.

The prefrontal cortex tissues of the experimental groups were examined for neuronal apoptosis by measuring the expression levels of Bax, caspase-3, and Bcl-2. According to the present findings, social isolation increased apoptosis (Figure 9). This was demonstrated by significantly increased levels of pro-apoptotic proteins caspase-3 [*p* < 0.0001; F (9, 40) = 38.02] and Bax [*p* < 0.0001; F (9, 40) = 14.31] and significantly decreased expression levels of the anti-apoptotic protein Bcl-2 [*p* < 0.0001; F (9, 40) = 20.65], compared to the control group. In contrast to the schizophrenic group, significantly low levels of Bax and caspase-3 were recorded in all treated groups, except for the risperidone group, which showed an insignificant decrease in Bax and caspase-3 intensity. Bcl-2 showed a significant increase in Na_2_SeO_3_ and EGCG groups and an insignificantly high level in other treated groups compared to the schizophrenic groups.

### 3.9 EGCG–SeNP treatment modifies the impact of social isolation on NMDARs in rats

**FIGURE 10:**
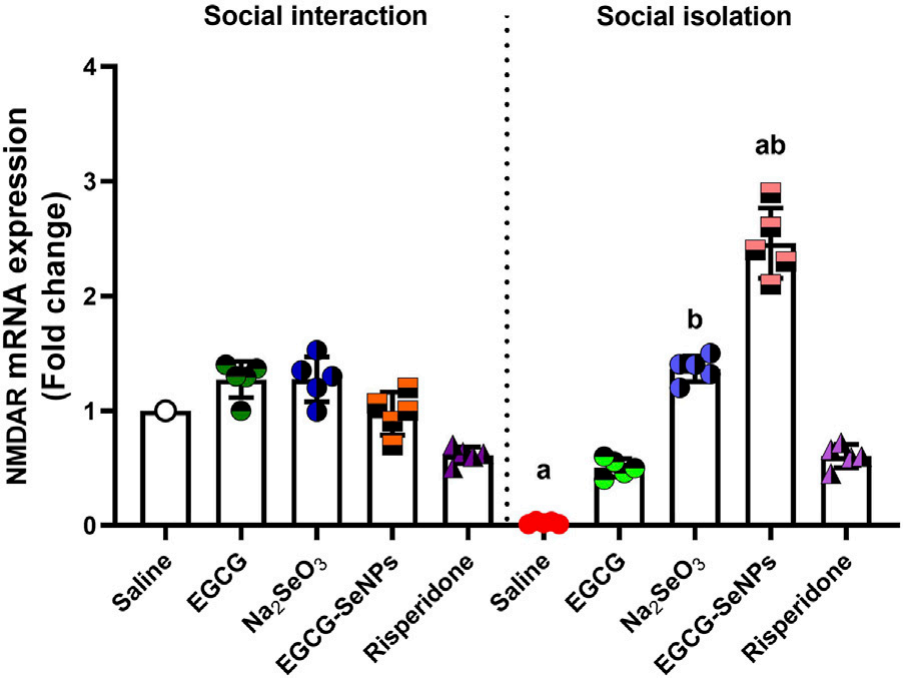
Effects of epigallocatechin-3-gallate (EGCG), sodium selenite (Na_2_SeO_3_), EGCG–SeNPs, and risperidone on the level of NMDAR mRNA expression in the prefrontal cortex of socially isolated rats**.** ^a,b^ denote significant differences (*p* < 0.05) compared with the control and isolated groups, respectively. All data are presented as the mean ± SD.

In the case of social isolation, a significant decrease in the prefrontal cortex’s NMDAR mRNA expression [*p* < 0.0001; F (9, 36) = 86.71] was noted compared to that in the control group (Figure 10). However, all treatments significantly increased NMDAR expression levels compared to isolated rats.

### 3.10 Effects of EGCG–SeNP treatment on histopathological alterations in the prefrontal cortical tissue of socially isolated rats

**FIGURE 11:**
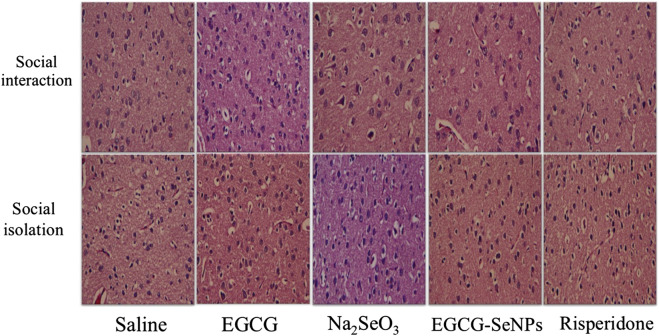
Effects of epigallocatechin-3-gallate (EGCG), sodium selenite (Na_2_SeO_3_), EGCG–SeNPs, and risperidone on histopathological changes in the prefrontal cortex tissue of socially isolated rats. Magnification = ×400.

In control untreated rats, the prefrontal cortex showed normal architecture with normal neurons and neuroglial cells, whereas in the social isolation group, neuronal loss and necrosis associated with pyknotic nuclei were observed. On the other hand, marked improvements were observed in the cellular structure of the prefrontal cortex in socially isolated rats treated with EGCG, Na_2_SeO_3_, EGCG-SeNPs, or risperidone (Figure 11).

### 3.11 Effect of EGCG on the D2 dopamine receptor

**FIGURE 12:**
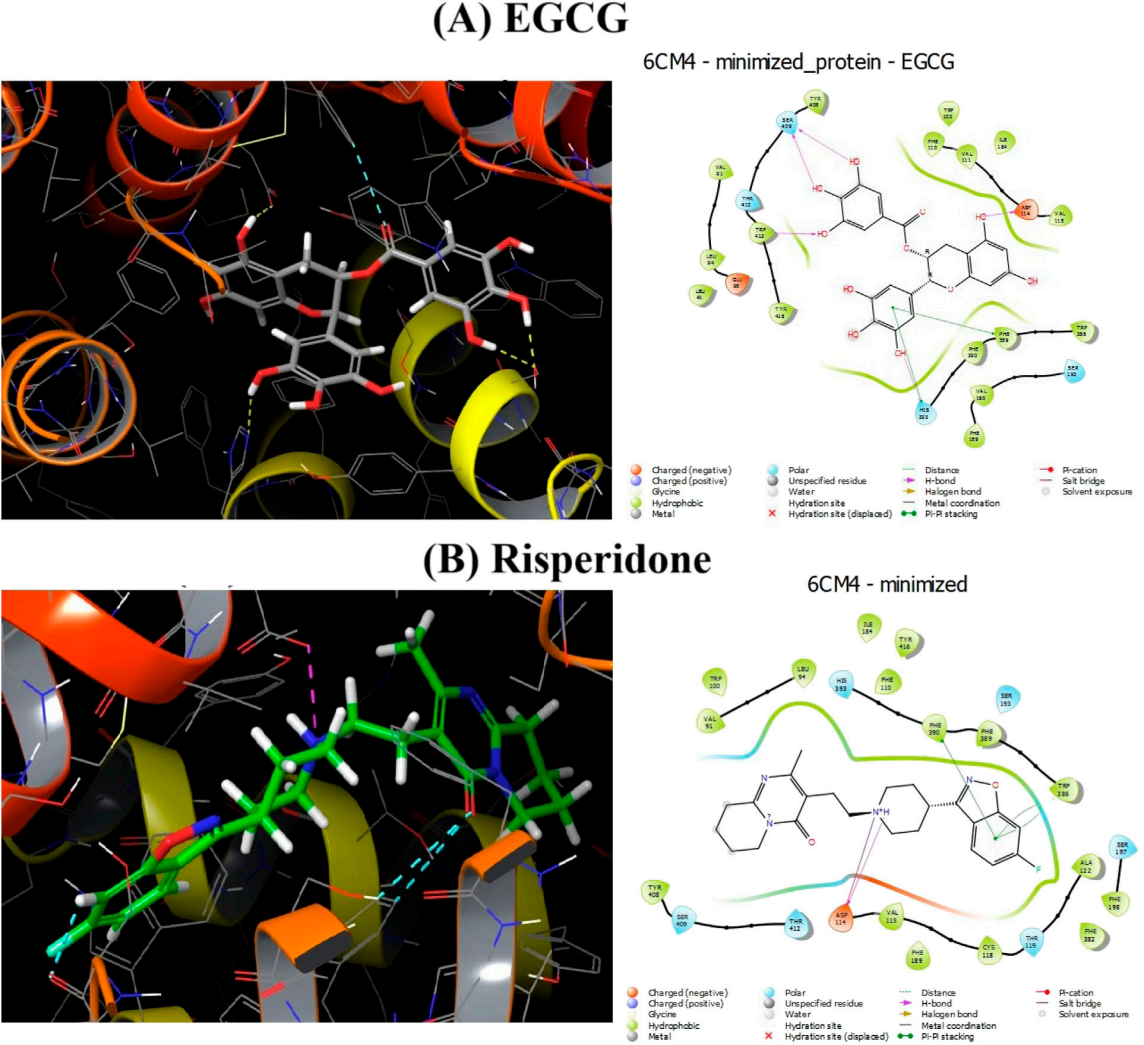
Docked 2D and 3D interactions between EGCG and risperidone, along with the 6cm4 protein. **(A)** EGCG. **(B)** Risperidone.

Docking experiments showed that EGCG was very effective at competing with the binding sites of the D2 dopamine receptor (DRD2) (Figure 12). The impact of the ligands (EGCG and risperidone) on DRD2 was examined using interaction-free energy. EGCG was able to dock at this receptor, as indicated by their H-interaction scores of −10.611 kcal/mol for DRD2–EGCG and −11.497 kcal/mol for DRD2–risperidone (Table 2).

**TABLE 2:** Docking study of ligands EGCG and risperidone with the D2 dopamine receptor (6CM4).

Ligand–receptor	Interaction type	Amino acid residue
Risperidone–6CM4	H donner Pi–pi interaction	Asp114 PHE390
EGCG–6CM4	H donner H acceptor Pi–pi interaction	SER 409/ASP 114 Trp 413 PHE 389/HIS 393

Abbreviations: Asp, aspartic amino acid; PHE, phenylalanine amino acid; SER, serine amino acid; Trp, tryptophan amino acid; HIS, histidine amino acid.

## 4 Discussion

The first-line evidence-based treatment for schizophrenia and other main psychotic illnesses is antipsychotic drugs. Every antipsychotic drug (first-generation and second-generation) has a distinct set of adverse effects that impact people in different ways. Significant extrapyramidal side effects are linked to first-generation antipsychotics, and significant weight gain and the emergence of metabolic syndrome are linked to second-generation antipsychotics (Stroup and Gray, 2018).

Nanomaterials have garnered increasing attention as potential co-therapeutic agents for a variety of disorders due to their superior bioavailability and improved medication distribution to specific tissues compared to existing drug delivery systems (Yuan et al., 2020).

In the present study, we evaluated the potential neuroprotective effects of EGCG–SeNPs against psychotic-like symptoms induced by social isolation in rats. According to our research, rats subjected to social isolation exhibited behavioral changes resembling those observed in schizophrenia. The present data demonstrated that social isolation increased the open-field locomotor activity of rats, which is consistent with the study by Xu et al. (2023). Moreover, our results align with those of Sestakova et al. (2013), who reported that the schizophrenic group had fewer rearing and grooming activities than the control group in the open-field test. The social interaction test revealed that social isolation also resulted in severe social deficiencies, as manifested by significantly high latency, few contacts with one another, and shorter interaction time, and this result is consistent with that of Nawwar et al. (2022). On the other hand, our results showed that EGCG–SeNPs reduced locomotor activity, slightly increased rearing and grooming activities, lowered latency to initial contact, and lengthened interaction times and contacts in open-field and social interaction tests. According to Chen et al. (2010), the behavioral performance of the stress control group was abnormal, whereas the behavioral performance of the EGCG-treated groups improved.

Current research demonstrated that social isolation led to oxidative imbalance in the prefrontal brain region, which is manifested by lowering levels of GSH, GR, SOD, and CAT and increasing lipid peroxidation, as shown by high MDA levels and NO synthesis. Additionally, levels of GPx were close between the isolated and control groups. The pathogenesis of schizophrenia is increasingly being linked to free radical-mediated CNS neuronal malfunction (Wu et al., 2013). However, only a small quantity of free radicals, such as superoxide ('O_2_^−^), hydroxyl ions ('OH), and nitric oxide (NO), are necessary for physiological processes; most of them are neutralized using cells’ antioxidant defense systems (Phaniendra et al., 2014). Excessive production of oxyradicals or a weak cellular antioxidant defense system can set off a series of reactions that can lead to cellular malfunction and even death (Chaudhary et al., 2023). Drechsel et al. (2012) and Das et al. (2015) also found comparable findings that social isolation altered the redox state, resulting in increased MDA and NO levels and decreased GSH, SOD, CAT, and GR levels, which, in turn, induced oxidative damage in the prefrontal cortex.

The social isolation model in rodents simulates how environmental disturbances affect cellular and molecular brain function, which is believed to be caused by cellular oxidative stress, where antioxidant defense systems are unable to balance and regulate endogenous ROS or RNS.

However, according to Jiang et al. (2013) and Be et al. (2020), EGCG–SeNP supplementation was able to inhibit social isolation-induced changes in the redox status of prefrontal brain tissue, as demonstrated by our research’s suppression of MDA and NO production and increase in GSH, SOD, CAT, GR, and GPx activities. These results support the promising neuroprotective and antioxidant properties of EGCG-SeNPs. The antioxidant effects of EGCG–SeNPs are attributable to both selenium, as stated by Yang and Yang, (2021), and, EGCG, which has been shown to increase GSH and SOD activities, as stated by Hsu et al. (2014).

The current study demonstrated that social isolation increases inflammatory cytokines, including TNF-α and IL-1β, as stated by Mumtaz et al. (2018). Our results concur with the findings of Omran et al. (2022), who reported that social isolation triggers the NF-κB signaling pathway, which increases pro-inflammatory cytokine transcription. The NF-κB family members, including NF-κB1, can form homodimers or heterodimers to produce the pleiotropic transcription regulator NF-κB. Once within the nucleus, activated NF-κB dimers promote the production of many pro-inflammatory cytokines, including TNFα and IL-1. ROS or neurotransmitters, such as glutamate, can activate it as a result of chronic stress from social isolation (Zlatković and Filipović, 2013). NF-κB expression was elevated in the PFC of individuals with schizophrenia (Long et al., 2022).

Our study found that EGCG–SeNPs reduced the level of NF-κB expression, along with TNF-α and IL-1β levels, considering the anti-inflammatory effects of selenium. This is consistent with the study by Yang and Yang (2021). Selenium exerts its physiological effects primarily through a family of proteins known as selenoproteins. Considerable attention has recently been directed toward SeNPs because of their excellent bioactivity, high bioavailability, and low toxicity (Yang and Yang, 2021). Selenite decreased inflammation via the prostaglandin E1 receptor in mice, according to Torres (2017). An earlier study by Bao et al. (2020) demonstrated that EGCG has anti-inflammatory properties, as observed by the significant decrease in IL-1β in mice, and showed that EGCG generated neuroprotective, anti-inflammatory, and cognitive deficit-improving effects. Our findings support the idea that EGCG also reduces inflammation by downregulating NF-κB, as reported by Mokra et al. (2022).

Although EGCG–SeNPs showed anti-inflammatory properties, it is essential to note that anti-inflammatory effects alone do not equate to antipsychotic efficacy. As stated by McCutcheon et al. (2020), schizophrenia is primarily associated with dysregulation in dopaminergic and glutamatergic neurotransmission, and current antipsychotic treatments mainly target dopamine D2 receptors. Therefore, the observed improvements in our model may reflect indirect benefits rather than a direct antipsychotic action.

Our findings support the fact that social isolation causes an abnormal neurochemical basis manifested by elevated prefrontal DA and 5-HT concentrations, as reported by Ago et al. (2013). Moreover, social isolation decreased glutamate levels in the cortex (Sestito et al., 2010; Shao et al., 2015). Our results are in accordance with Cale et al. (2024), who demonstrated that GABA concentration was also reduced. Furthermore, our findings were consistent with those of Ali et al. (2017), who showed that prolonged social isolation causes neurological degeneration in the brain, as indicated by a significant increase in brain AChE activity compared to the corresponding socially housed control group. According to Mumtaz et al. (2018), growing data have strongly indicated that, in both people and animals, social isolation serves as a trigger for changes in social behavior, response to stress, the neuroendocrine and neurochemical systems’ function, and anatomical, physiological, and behavioral changes. It has been suggested that acute or chronic social isolation may exhibit indications of neurological and mental conditions such as schizophrenia. Neurotransmitters, including dopamine, serotonin, glutamate, GABA, and the nitrergic system, are all affected by social isolation. The pathophysiological effects of social isolation include cholinergic system dysfunction, oxidative stress-mediated mitochondrial dysfunction, and inflammatory factors.

In contrast to the schizophrenia group in our study, EGCG–SeNPs modulated the neurotransmitters by increasing glutamate and GABA and decreasing SE, DA, and AChE. EGCG–SeNP treatment in the current research showed anti-schizophrenic efficacy by modifying monoaminergic and cholinergic transmission after social isolation. According to Solovyev (2015), it is likely that selenium and selenium-containing proteins influence at least a few neurotransmission routes. Additionally, selenium appears to be utilized by dopaminergic pathways due to increased reactive oxygen species, and selenium proteins seem to have a special function in the development and activation of GABAergic neurons. Additionally, Refaay et al. (2022) claimed that certain compounds of organoselenium have an effect on Alzheimer’s disease by inhibiting AChE. SeNPs reduce AChE activity, suggesting that inflammation has been reduced (Ebokaiwe et al., 2020). Our findings concur with those of Nakagawa et al. (2019), demonstrating that the social isolation group’s prefrontal cortex BDNF concentration was noticeably lower than that of the control group.

Dopaminergic, cholinergic, and serotonergic neurons in the central nervous system (CNS) are all affected by BDNF, a mediator of neuronal survival and plasticity. BDNF is aberrantly regulated in the central nervous system of animal models of schizophrenia, and it has been observed that fewer BDNF-positive neurons are present in schizophrenia patients (Ahmad et al., 2023). We observed that EGCG–SeNPs increased the BDNF levels. These results align with the findings of Ding et al. (2017), who confirmed that BDNF mRNA levels were elevated following EGCG administration.

We found that corticosterone levels increased in cases of social isolation, as documented by Colaianna et al. (2013). According to Chen et al. (2022), postnatal stress increased blood corticosterone levels in humans and rats. Our study demonstrated that EGCG–SeNPs reduced corticosterone levels following elevated levels induced by social isolation. An et al. (2023) indicated that supplementing selenium inhibits corticosterone from aggravating endoplasmic reticulum stress and compromising insulin signaling.

Additionally, in our research, social isolation increased apoptosis in the prefrontal cortex, which decreased the expression of the anti-apoptotic protein Bcl-2 and increased the expression of the pro-apoptotic proteins Bax and caspase-3, as stated by Xu et al. (2023). We noticed that treatment with EGCG–SeNPs suppresses apoptotic processes linked to social isolation, which increased the expression of the anti-apoptotic protein Bcl-2 and decreased the expression of pro-apoptotic proteins Bax and caspase-3.

According to Nan et al. (2018), through the PI3K/AKT/eNOS signaling pathway, epigallocatechin-3-gallate lowers neuronal apoptosis in rats following middle cerebral artery occlusion injury.

Selenium is not only a critical component of the brain’s antioxidant system but has also been shown to reduce oxidative stress in the brain by regulating apoptosis, mitochondrial biogenesis, and Ca^2+^ channels (Ebokaiwe et al., 2020).

The present study revealed that social isolation reduced the PFC levels of the NMDA. Our findings align with those of Nakazawa and Sapkota, (2019), who confirmed that NMDAR hypofunction occurs in GABAergic neurons in the early postnatal period in schizophrenia, where the pathophysiology of schizophrenia is significantly influenced by NMDAR hypofunction. In a previous study by Hermes et al. (2010), social isolation significantly reduced the PFC levels of the NMDA receptor subunit NR1 in female rats. Moreover, social isolation alters the sensitivity of the NMDA receptor (Mumtaz et al., 2018), whereas our findings indicate that EGCG–SeNPs help increase their concentration.

However, it is critical to recognize the limitations of the social isolation model, although it is a commonly used paradigm in preclinical research to cause behavioral and neurochemical changes that mimic some features of schizophrenia. Social isolation does not adequately represent the complex pathophysiology of schizophrenia; rather, it largely simulates the negative symptoms and cognitive deficits observed in the disease. Additionally, Jiang et al. (2013) claimed that social isolation uses oxidative stress mechanisms, specifically impacting cortical interneurons, causing schizophrenia-like symptoms in rodents. Furthermore, social isolation affects molecular markers of neuroplasticity, which are similarly linked to schizophrenia, according to Be et al. (2020).

## 5 Conclusion

As evidenced by the current findings, EGCG–SeNPs reduced psychotic-like symptoms against social isolation-mediated schizophrenia in rats, which enhanced monoaminergic and cholinergic transmission and restored the excitatory–inhibitory amino acid imbalance. Additionally, it decreased inflammation, restored the apoptotic balance, and enhanced the antioxidant capacity of the prefrontal cortex tissue and rats’ behavioral test performance. These effects relate to neuromodulatory effects on neurotransmitter activity. Therefore, EGCG–SeNPs show potent neuroprotective and antipsychotic effects against social isolation-induced schizophrenia. This highlights the therapeutic potential of EGCG–SeNPs and aligns with the growing body of research supporting the efficacy of natural products and nanomaterials in the treatment of mental illness. Future research should incorporate dose-response studies to determine the best therapeutic ranges and assess safety thresholds, even though this study concentrated on a single effective dose (0.5 mg/kg). However, additional studies are required to validate the effects, safety, and efficacy of EGCG–SeNPs for the long term.

## Data Availability

The original contributions presented in the study are included in the article/supplementary material; further inquiries can be directed to the corresponding author.
